# Subthalamic and pallidal deep brain stimulation for Parkinson’s disease—meta-analysis of outcomes

**DOI:** 10.1038/s41531-021-00223-5

**Published:** 2021-09-06

**Authors:** M. Lenard Lachenmayer, Melina Mürset, Nicolas Antih, Ines Debove, Julia Muellner, Maëlys Bompart, Janine-Ai Schlaeppi, Andreas Nowacki, Hana You, Joan P. Michelis, Alain Dransart, Claudio Pollo, Guenther Deuschl, Paul Krack

**Affiliations:** 1 Department of Neurology, Inselspital, Bern University Hospital, University of Bern, Bern, Switzerland; 2 Aleva Neurotherapeutics SA, Lausanne, Switzerland; 3 Department of Neurosurgery, Inselspital, Bern University Hospital, University of Bern, Bern, Switzerland; 4 Department of Neurology, UKSH, Christian-Albrechts-University, Kiel, Germany

**Keywords:** Outcomes research, Parkinson's disease

## Abstract

Although deep brain stimulation (DBS) of the globus pallidus internus (GPi) and the subthalamic nucleus (STN) has become an established treatment for Parkinson’s disease (PD), a recent meta-analysis of outcomes is lacking. To address this gap, we performed a meta-analysis of bilateral STN- and GPi-DBS studies published from 1990-08/2019. Studies with ≥10 subjects reporting Unified Parkinson’s Disease Rating Scale (UPDRS) III motor scores at baseline and 6–12 months follow-up were included. Several outcome variables were analyzed and adverse events (AE) were summarized. 39 STN studies (2035 subjects) and 5 GPi studies (292 subjects) were eligible. UPDRS-II score after surgery in the stimulation-ON/medication-OFF state compared to preoperative medication-OFF state improved by 47% with STN-DBS and 18.5% with GPi-DBS. UPDRS-III score improved by 50.5% with STN-DBS and 29.8% with GPi-DBS. STN-DBS improved dyskinesia by 64%, daily OFF time by 69.1%, and quality of life measured by PDQ-39 by 22.2%, while Levodopa Equivalent Daily Dose (LEDD) was reduced by 50.0%. For GPi-DBS information regarding dyskinesia, OFF time, PDQ-39 and LEDD was insufficient for further analysis. Correlation analysis showed that preoperative L-dopa responsiveness was highly predictive of the STN-DBS motor outcome across all studies. Most common surgery-related AE were infection (5.1%) and intracranial hemorrhage (3.1%). Despite a series of technological advances, outcomes of modern surgery are still comparable with those of the early days of DBS. Recent changes in target selection with a preference of GPi in elderly patients with cognitive deficits and more psychiatric comorbidities require more published data for validation.

## Introduction

The first implantation of deep brain electrodes for tremor in Parkinson’s disease (PD) by Alim-Louis Benabid in the late 1980s in the thalamic ventral intermediate nucleus^1^ paved the way for the worldwide application of deep brain stimulation (DBS) in PD. Despite an initial lack of randomized controlled studies, DBS of the subthalamic nucleus (STN) or the internal part of the globus pallidus (GPi) quickly became a well accepted therapy for advanced PD with motor complications due to its convincing effect on motor symptoms, shown by smaller and uncontrolled studies^2–9^ and a highly quoted and influential meta-analysis by Kleiner-Fisman and colleagues^10^. Since then, the field of DBS has undergone important technical progress, and the efficacy and safety of bilateral STN- and GPi-DBS has been underlined by large randomized controlled trials (RCT)^11–18^ and many additional uncontrolled studies, while the choice of the best target remains a matter of controversial debate. This is partly explained by the fact that the results of the existing RCTs are difficult to compare due to their different primary outcomes such as quality of life^13,15,17^, time in ON without dyskinesia measured with patient diaries^14,16^, motor symptoms (UPDRS-III)^11,12^, and functional health measured with a composite score for cognitive, mood, and behavioral effects^18^. Fortunately, most of the RCTs also use some common scales either as primary or secondary outcomes. Importantly, all studies use the UPDRS, but when comparing UPDRS-III (whether primary or secondary endpoint), outcomes are highly variable across published randomized controlled studies.

Although several meta-analyses have been published in recent years, these focused either only on the outcome of specific symptoms or on RCTs^19–21^ and therefore include only a limited number of patients. Due to the different endpoints of the studies, the inconsistent reporting of symptoms such as dyskinesias or missing important outcome parameters, such as quality of life, these meta-analyses had to be basically limited to an analysis of motor outcome (UPDRS III) as a common secondary endpoint. Ultimately, both RCTs and previous meta-analyses leave many questions unanswered because of the aforementioned limitations. Our meta-analysis represents the first since the work of Kleiner-Fisman et al.^10^ to include the majority of available STN and GPi studies, allowing not only to add analysis of published outcomes from the last 15 years, but also maximizing the number of subjects included, and analyzing multiple outcome parameters simultaneously.

## Results

### Literature review

As shown in Supplementary Fig. 1, the literature search identified 256 original citations. Sixty-one articles were selected according to the inclusion criteria. Twenty-seven contributed to both safety and efficacy, 20 contributed to safety analysis only, and 12 contributed to efficacy analysis only. A detailed list of the corresponding articles is provided in Supplementary Table 1. Thirty-four articles met efficacy inclusion criteria, to which five additional articles, identified by P.K. and L.L., were added. These historical papers (for more detail see supplementary material) were added to facilitate the understanding of the existing literature without any relevant change in the statistical results. After this process, 39 STN studies^2,3,11–13,15–18,22–51^ involving 2035 subjects with follow-up data (T1) for 1747 subjects and 5 GPi studies^2,11,12,18,30^ with 292 subjects (*n* at T1 = 291) were available for efficacy outcome analysis. For safety, 47 articles reporting on a total of 2818 enrolled subjects met the inclusion criteria.

### Patient characteristics

Detailed patient characteristics and demographic information are shown in Supplementary Table 2. Study sample sizes (some papers reporting outcomes of both STN and GPi DBS) ranged from 10 to 299 (median [range] = 33) and follow-up time considered for this analysis spanned from 6 months to 2 years (weighted mean, 13.1 ± 5.9 months). The weighted mean age at surgery was 59.1 ± 2.9 years (range of means, 50.7–66.7). Weighted average disease duration prior to surgery was 12.2 ± 2.1 years (range of means, 7.3–19.0). Compared to the period covered by Kleiner-Fisman et al. (1993–2005)^10^, subjects of STN studies since 2005 were less affected at baseline based on UPDRS-II and -III scores (Table 1 and Supplementary Table 3).

**Table 1:** Patient characteristics in relation to the publication period.

Publication year & therapeutic target	STN 1993–2004					STN 2005–2019				GPi 2005–2019		
	Weighted mean at baseline	Difference between postoperative stim ON/med OFF and preoperative med OFF			Weighted mean at baseline	Difference between postoperative stim ON/med OFF and preoperative med OFF			Weighted mean at baseline	Difference between postoperative stim ON/med OFF and preoperative med OFF		
		Pooled mean estimate	95% CI lower limit	95% CI upper limit		Pooled mean estimate	95% CI lower limit	95% CI upper limit		Pooled mean estimate	95% CI lower limit	95% CI upper limit
Age at operation (years)	58.52	-	58.30	58.74	59.06	-	58.91	59.21	60.65	-	52.04	69.27
Disease duration (years)	14.21	-	14.08	14.34	11.81	-	11.71	11.91	11.43	-	9.80	13.05
UPDRS II	25.82	13.35 (51.7%)	10.85 (42.0%)	15.85 (61.4%)	21.57	8.79 (40.9%)	6.66 (31.0%)	10.91 (50.8%)	19.19	3.55 (18.5%)	2.41 (12.6%)	4.68 (24.4%)
UPDRS III	49.35	27.55 (55.8%)	24.23 (49.1%)	30.87 (62.6%)	42.55	20.93 (49.5%)	18.78 (44.4%)	23.07 (54.6%)	42.65	12.13 (28.4%)	9.73 (22.8%)	14.53 (34.1%)

### Analyses of heterogeneity and bias

Cochran’s Q test revealed statistically significant evidence for heterogeneity between STN studies in the datasets collected for UPDRS-II and -III score (*P* < 0.0001), dyskinesia (*P* < 0.0013), daily OFF time, LEDD (*P* < 0.0001), and for improvement of PDQ-39 SI (*P* = 0.0008). Between the GPi studies, there was no significant evidence of heterogeneity for UPDRS-II (*P* = 0.5916) and -III (*P* = 0.4321) scores, possibly due to the small sample size, while the information for dyskinesia, daily OFF time, PDQ-39 SI, and LEDD was insufficient for further analysis.

Funnel plots and Egger’s regression test revealed statistically significant asymmetrical distribution only for the PDQ-39 outcome with STN-DBS (*P* = 0.0028) (Supplemantary Fig. 2 and Supplementary Table 4), which is due to a variation of the effect observed in smaller studies because of their sample size and might indicate publication bias.

### UPDRS-II

**Fig. 1:**
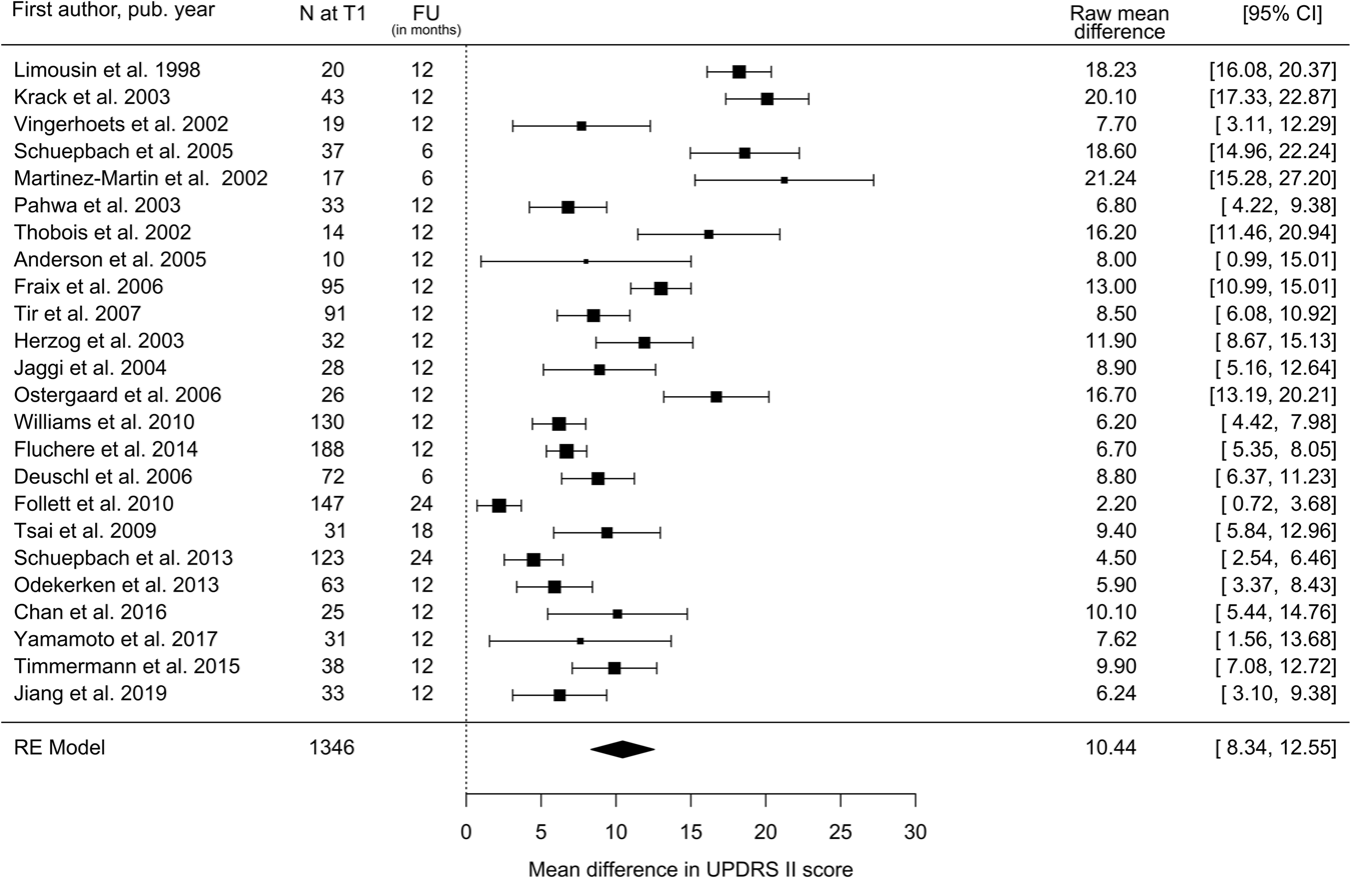
Estimated decrease in total UPDRS II score following STN-DBS. Postoperative stimulation-ON/medication-OFF vs. preoperative OFF-medication state: UPDRS II Mean Difference. *N* = number of subjects at follow-up; FU = follow-up time; CI = confidence interval; RE Model = random-effects model.

Twenty-four STN-DBS studies with a total of 1346 subjects reported an estimated decrease in total UPDRS-II score at follow-up in the stimulation-ON/medication-OFF state compared with the preoperative OFF-medication state of 10.4 points (95% CI: 8.3–12.6; Fig. 1), equivalent to a 47% (95% CI: 37.4–56.7%) reduction. The extent of this decrease in UPDRS-II score showed a dose–response relationship with preoperative response to the L-Dopa challenge (Supplementary Fig. 3). Compared to the period covered by Kleiner-Fisman et al. (1993–2005)^10^, the STN studies since 2005 have shown a slightly lower response of UPDRS-II to DBS (49.9% vs. 40.9%, Table 1 and Supplementary Table 3).

**Fig. 2:**
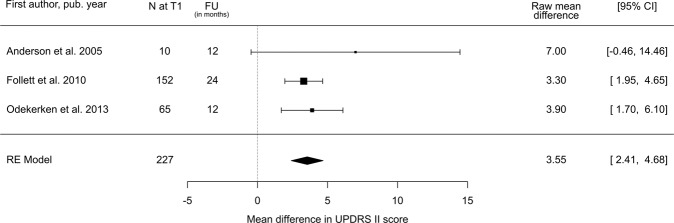
Estimated decrease in total UPDRS II score following GPi-DBS. Postoperative stimulation-ON/medication-OFF vs. preoperative OFF-medication state: UPDRS II Mean Difference. *N* = number of subjects at follow-up; FU = follow-up time; CI = confidence interval; RE Model = random-effects model.

For GPi, three studies with a total of 227 subjects were reporting a decrease in UPDRS-II scores in medication-OFF condition of 3.6 points (95% CI: 2.4–4.7), equivalent in percentage to a change of 18.5% (95% CI: 12.6–24.4%; difference between postoperative stimulation-ON/medication-OFF state and preoperative OFF-medication state; Table 1 and Fig. 2).

### UPDRS-III

**Fig. 3:**
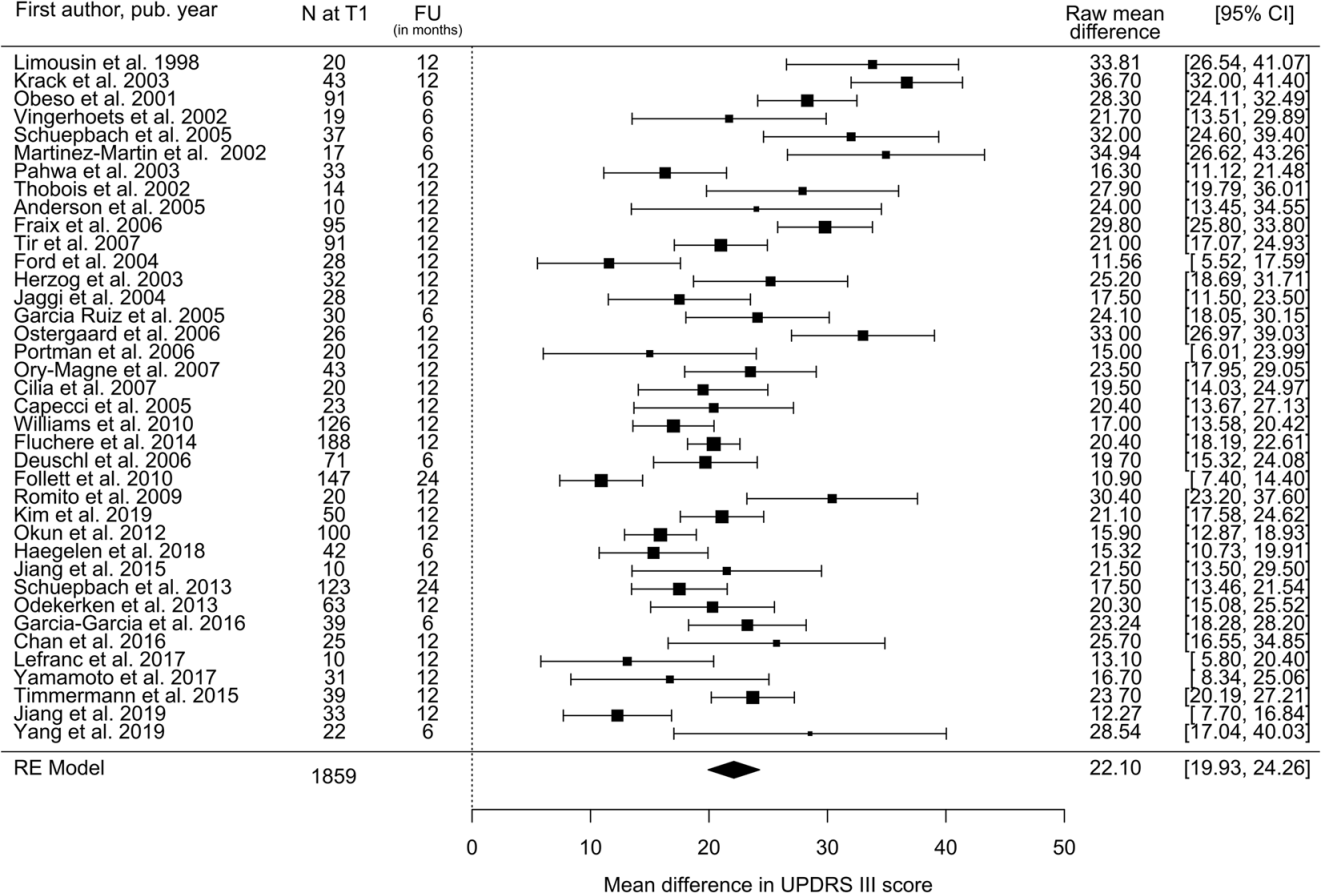
Estimated decrease in total UPDRS-III score following STN-DBS. Postoperative stimulation-ON/medication-OFF vs. preoperative OFF-medication state: UPDRS III Mean Difference. *N* = number of subjects at follow-up; FU = follow-up time; CI = confidence interval; RE Model = random-effects model.

**Fig. 4:**
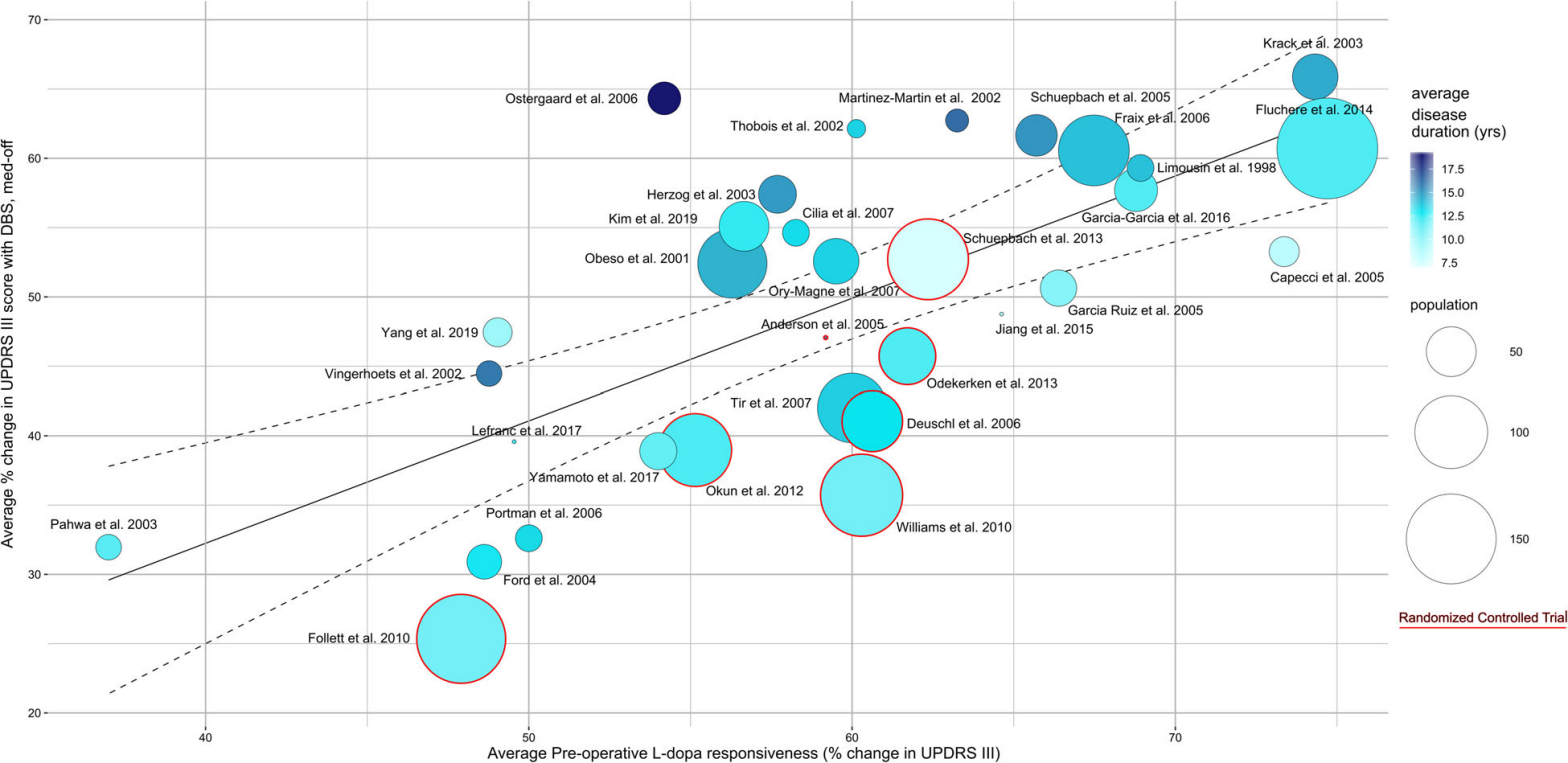
Preoperative L-dopa response predicts STN-DBS motor outcome. Dose-response relationship between preoperative L-dopa response and improvement in UPDRS-III after STN-DBS, considering average disease duration (color shade), study population size (circle diameter), and randomized controlled trials (red frame). Studies reviewed indicated by first author and year of publication.

**Fig. 5:**
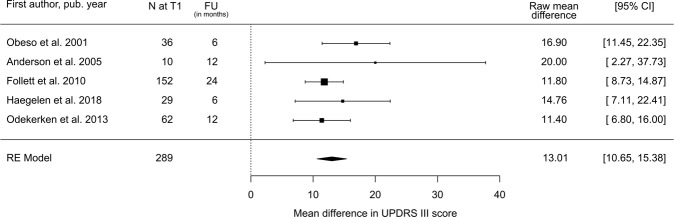
Estimated decrease in total UPDRS-III score following GPi-DBS. Postoperative stimulation-ON/medication-OFF vs. preoperative OFF-medication state: UPDRS III Mean Difference. *N* = number of subjects at follow-up; FU = follow-up time; CI = confidence interval; RE Model = random-effects model.

Thirty-eight STN studies comprising 1859 subjects reported change in UPDRS-III score and estimate of standard error. The estimated decrease in UPDRS-III score at follow-up compared to baseline was 22.1 points (95% CI: 19.9–24.3; Fig. 3), equivalent to a 50.5% (95% CI: 45.6–55.5%) reduction. The magnitude of decrease in UPDRS-III with STN stimulation showed a dose–response relationship with preoperative response to the L-Dopa challenge (Fig. 4). Since 2005, studies have shown a slightly lower response of UPDRS-III to STN-DBS compared to the period covered by Kleiner-Fisman et al. (1993–2005)^10^ (Table 1 and Supplementary Table 3). According to our literature search criteria, only five GPi studies with 289 subjects were available, which showed a decrease in UPDRS-III score after surgery of 13.0 points (95% CI: 10.7–15.4; Fig. 5), equivalent to a reduction of 29.8%.

### Dyskinesia

**Fig. 6:**
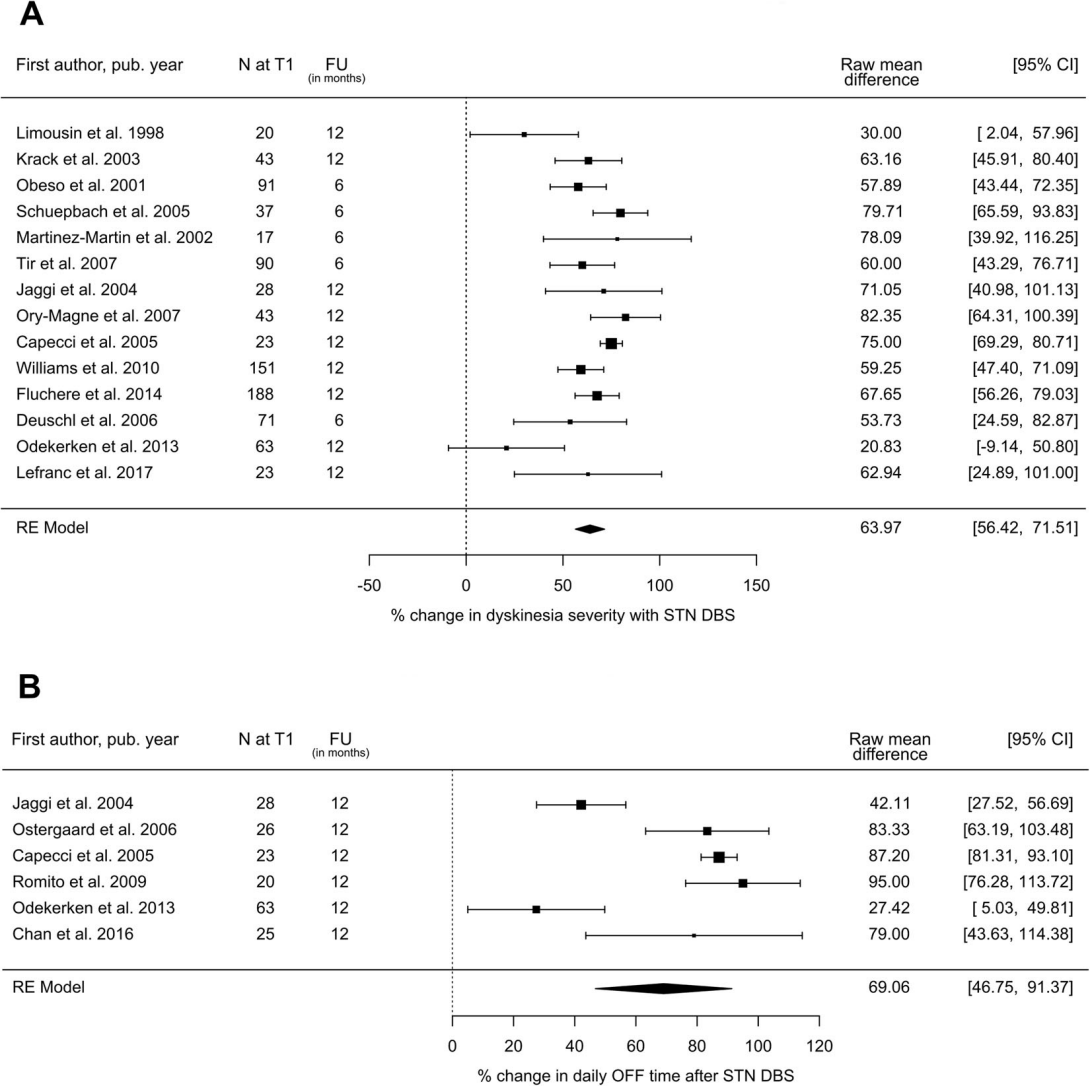
Change in motor complications following STN-DBS. Postoperative vs. preoperative state: change in mean dyskinesia severity (**A**) and mean OFF time (**B**). *N* = number of subjects at follow-up; FU = follow-up time; CI = confidence interval; RE Model = random-effects model.

Data for dyskinesias were very heterogeneous between studies, due to use of different rating scales. However, conversion of mean study scores to percentages relative to their respective scales allowed for comparison. For this purpose, only scales that provide an overall assessment of dyskinesias such as UPDRS-IV (item 32 to 35)^52^, the Abnormal Involuntary Movement Scale^53^, and the Marconi Dyskinesia Rating Scale^54^ were used. Patient diaries and individual items of the UPDRS IV could not be included as these assess either only the duration or the severity of dyskinesias and therefore do not allow such a conversion. For fourteen STN studies with 950 subjects, the average reduction in dyskinesia at follow-up could be determined and was 64.0% (95% CI: 56.4%–71.5%; Fig. 6A). For GPi-DBS there was a weighted mean improvement of 39.7% per Obeso et al.^2^ (*n* = 38) and Odekerken et al.^18^ (*n* = 62). Since only one of the included GPi studies^2^ provided the required data quality with standard deviation, a pooled estimate could not be performed.

### Daily OFF time

Only six STN studies with 185 subjects provided information on the change in daily OFF time (item 39 of UPDRS-IV or patient diaries) and the estimation of the standard error. After converting the average study values into percentages relative to their respective scales, the comparison revealed an average decrease of 69.1% (95% CI: 46.8%–91.4%; Fig. 6B), while the information for GPi was not sufficient for further analysis.

### Quality of life

**Fig. 7:**
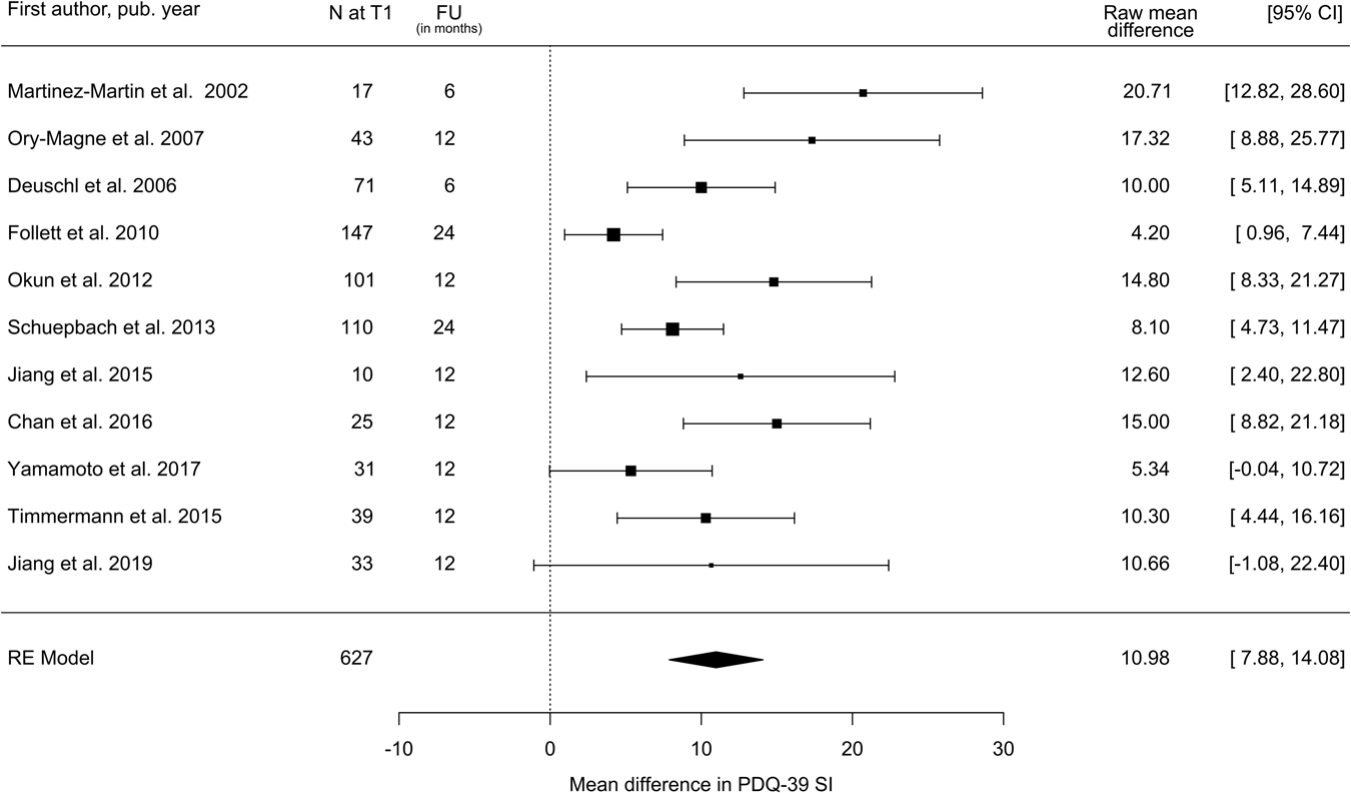
Change in total PDQ-39 summary index (SI) score following STN-DBS. Postoperative vs. preoperative state: PDQ-39 (SI) Mean Difference. *N* = number of subjects at follow-up; FU = follow-up time; CI = confidence interval; RE Model = random-effects model.

Change in quality of life was assessed with the PDQ-39 SI^55^ in eleven STN studies with 627 subjects. An average improvement of the summary index score of 11.0 points (95% CI: 7.9–14.1; Fig. 7) at follow-up (approx. 22.2% of baseline weighted mean) was observed, while the information for GPi was not sufficient to perform the same analysis.

### Levodopa equivalent daily dose (LEDD)

Thirty-one STN studies with 1644 subjects provided information regarding changes in LEDD with an average LEDD reduction after surgery of 50.0% (95% CI: 45.1%–54.8%; Supplementary Fig. 4), while the information for GPi was not sufficient to perform the same analysis.

### Adverse events (AE)

Safety data were included from forty-seven STN and four GPi studies. The median proportion of surgery-related intracranial hemorrhage (ICH) was 3.1% including both target structures (3.45% for STN-DBS, 2.2% for GPi-DBS). The median proportion of surgery-related ICH with reported permanent neurological deficits was 1.65%. Some studies additionally distinguished ICH with temporary deficits and asymptomatic ICH with a median proportion of 1.0% and 2.1%, respectively. The median proportion of surgery-related infections was 5.1%. A detailed list of the proportions of surgery-, hardware-, disease-, stimulation-, and therapy-related side effects is provided in Supplementary Table 5.

### Prediction of STN-DBS benefit

For thirty-two studies, the change in UPDRS-III score caused by L-dopa challenge at baseline (T0) was comparable to that achieved under STN-DBS only (medication-OFF). Graphical representation of this analysis is presented in Fig. 4. Unfortunately, the data available from our article pool for GPi-DBS did not allow us to conduct the same analysis for this therapeutic target.

To evaluate the possible predictive power of the pre-operative change for post-operative results of STN-DBS, normality of the datasets collected for both variables was evaluated through a Shapiro–Wilk test (*P* = 0.6673: *P* = 0.2569). A Pearson correlation test was then performed that revealed a statistically significant correlation between UPDRS-III change at baseline upon L-dopa challenge and UPDRS-III change (medication-OFF) after STN-DBS: correlation coefficient = 0.689958 (*P* = 0.00001).

### Variables affecting prediction of benefit from STN-DBS

To identify factors likely to influence the quality of the prediction made by the change in UPDRS-III score upon pre-operative L-dopa challenge, the ratio of UPDRS-III change post-op/UPDRS-III pre-op L-dopa (“surgical efficiency index“) was used as a proxy for assessing the quality of a prediction (if >1, post-op benefit was underestimated by L-dopa challenge; if <1, post-op benefit was overestimated; if=1, perfect prediction). Correlation between this ratio (calculated for each study) and several variables was evaluated through a Pearson correlation test (if both datasets followed a normal distribution) or Spearman test (if one dataset did not follow a normal distribution).

From our correlation analysis, the average disease duration prior to implantation (*R*^2^ «1, Supplementary Fig. 5) appears to be the only measured preoperative variable of statistically significant influence on the predictive power of the pre-operative L-dopa challenge (*P* = 0.003) (Supplementary Table 6).

No statistically significant correlation was observed between the motor benefit prediction ratio and any other measured individual variable (Supplementary Table 6).

## Discussion

The aim of this article was to provide an updated review of the published literature on the outcomes of bilateral STN- and GPi-DBS for PD. According to our inclusion criteria, a majority of studies focused on the STN (*n* = 39) involving 1747 subjects with follow-up data and, to a much lesser extent, the GPi (*n* = 5) with 291 subjects with follow-up data. Considering that the question of whether STN- or GPi-DBS is clinically superior is still a matter of controversial debate, and given our efforts to minimize biases introduced by the data retrieval and analysis method (e.g., use of random-effects model, standardized literature search, and data extraction protocol), the striking numerical predominance of the STN studies over the GPi studies is very surprising. GPi is an easier target as far as immediate postoperative management is concerned. Unlike STN-DBS, GPi-DBS does not require fine-tuning between stimulation intensity and medication dosage. Unilateral surgery can be easily proposed for GPi, as there is no deterioration of the non-operated side in relation to postoperative medication reduction^56^. After the publication of the US Veterans Administration study comparing STN versus GPi-DBS, a triumphant return of pallidal stimulation was predicted^57^. Indeed, there has been a shift towards implantation of GPi based on understanding of the outcomes of the VA study^11^. GPi targeting increased and was selected for older patients with poorer cognitive and mood indices^58^. Our meta-analysis of the literature shows that this new popularity of GPi as a target has not yet translated into publications addressing GPi-DBS and justifying such a gradual shift in patient selection. Future randomized controlled trials are needed to evaluate the impact on quality of life when recruiting patients with such selection bias, as surgical complications increase with age^39^, depression and cognitive decline are important determinants of quality of life in PD^59^, and a previous study had shown that in PD patients with preoperatively borderline impaired cognition, STN-DBS did not provide any benefit in terms of quality of life^60^.

Even 14 years after the meta-analysis by Kleiner-Fisman et al.^10^, which provided the first comprehensive results to estimate the extent of the effects of STN-DBS in PD, studies reporting the outcomes for the main indication of DBS, namely motor fluctuations and dyskinesias, are still very heterogeneous, due to missing reporting or more importantly, different evaluation tools (UPDRS items in different combinations, different specific rating scales, or patient diaries). As a result, the available and comparable data are limited, especially with regard to dyskinesia, and, at least in the context of our analysis, only allow a comparison of percentage changes in different specific total dyskinesia scores, but not of dyskinesia severity or dyskinesia duration only. This highlights the urgent need for a uniform and more detailed assessment of these motor complications across centers. On average, dyskinesia and daily OFF time were significantly improved by STN stimulation (62.5% decrease in dyskinesia, *n* = 950; 69.1% decrease of OFF time), whereas the available data for GPi-DBS was not sufficient for a comprehensive analysis, but at least showed a weighted mean improvement of 39.7%. In contrast, change in UPDRS-III scores in the stimulation-ON/medication-OFF condition compared to the baseline medication-OFF condition is available throughout almost all publications and therefore still the most commonly used measure to assess DBS efficacy. On average, there was a convincing improvement in UPDRS-III scores by STN- and GPi-DBS compared to preoperative baseline. Although we have not conducted a comparative analysis of these two targets and the discrepant counts of STN and GPi studies affects the precision of our estimates, the improvement in motor symptoms (UPDRS-III) of 50.5% for STN-DBS versus 29.8% for GPi-DBS suggests that STN-DBS is superior to GPi-DBS in terms of motor outcomes.

Interestingly, despite technical advances, STN studies showed a slightly lower response to DBS in the period 2005–2019 compared with the period 1993–2004 covered by Kleiner-Fisman et al.^10^, which may be due to the fact that subjects enrolled in the studies since 2005 had a shorter disease duration and were less affected in terms of UPDRS-II and -III. In addition, this may also be explained by the fact, that the technical achievements in imaging, targeting, and intraoperative procedures have not yet translated into a relevant advantage over the reviewed period, as they have not all been consistently used by all centers.

The question of the optimal DBS target in PD may not be sufficiently clarified with the UPDRS-III medication OFF score as the primary endpoint, because it does not take into account non-motor symptoms. Results of the two target structures are also difficult to compare, as both targets require very different postoperative management. A relevant LEDD reduction is only possible with STN-DBS and also mandatory to reduce dyskinesia. This, in turn, can lead to apathy and dopamine withdrawal syndrome on one side, or to an improvement of impulse control disorders on the other side with a major impact on quality of life^61^. Therefore, quality of life assessed by the PDQ-39 may be more appropriate to assess DBS efficacy, as this outcome measure is affected not only by changes in motor symptoms but also by changes in non-motor symptoms and motor complications. For this reason, QOL measurement was introduced in DBS^62^ and was chosen as the main outcome criterion in the very first randomized controlled trial on DBS^13^. Our meta-analysis showed a moderate improvement in quality of life (PDQ-39) of approximately 22% with STN-DBS, while the information for GPi was not sufficient for analysis. Although there were only a few STN studies (*n* = 11) with high variance of results, this improvement is clinically highly relevant, when considering that patients with best medical treatment tend to worsen their quality of life over the same period^61^. Activities of daily living (UPDRS-II) as a main determinant of quality of life improved on average by 47% after STN surgery, while the GPi studies showed a lower improvement of 18.5%. However, due to the relatively few GPi studies that qualified for analysis, this finding must be interpreted with caution, in particular considering the fact that three years after DBS surgery there was no difference in long-term results between STN and GPi with regard to quality of life^63–65^.

Overall, the published studies are heterogeneous, especially regarding the (primary) outcome parameters, the assessment tools used, and the selection criteria for DBS, which can certainly be explained in part by our inclusion criteria and the inclusion of small non-randomized open-label studies. However, this heterogeneity also affects the RCTs as there are differences in the choice of primary outcome parameters, partly due to the different requirements of the regulatory authorities in the United States and Europe. Furthermore, patient selection is not identical as far as levodopa-sensitivity is concerned (Fig. 4). Postoperative management can be impacted by differences in healthcare systems, which can determine whether DBS is managed on an outpatient or an in-hospital basis with consequences on potential expert time devoted to an individual patient. Therefore, we believe that this heterogeneity reflects clinical reality. Many questions including target preference (STN vs GPi) could not be solved so far by randomized controlled trials, which did not come to identical conclusions^11,18^, resulting in DBS targeting (STN vs GPi) based on experience of a team, differences in health care systems, and analysis of the full literature, rather than just evidence-based medicine. Therefore, our meta-analysis highlights the need to identify predictors of motor and non-motor outcomes or to investigate the impact of new techniques, such as the advent of newer imaging and targeting techniques, MRI-guided asleep DBS, current steering, or closed loop DBS. To address such knowledge gaps, the use of prospective registries with targeted data collection in the pre-operative, operative, and post-operative phases of DBS treatment^66^ may be a good option providing larger data allowing for more detailed analyses^67^.

Although the efficacy of DBS for the treatment of PD is well recognized, preoperative predictive factors for a favorable outcome of DBS are still not sufficiently known. In accordance with Kleiner-Fisman et al.^10^, the preoperative L-dopa responsiveness was highly predictive of the motor outcome of STN-DBS (Pearson *P* = 0.00001). From our correlation analysis, the average disease duration prior to implantation appears to be influencing the quality of the prediction made by pre-operative-L-dopa challenge for the benefit of STN-DBS (*P* = 0.003). The longer the patient is suffering from PD before the implantation, the more the prediction made by pre-operative L-dopa response will correspond to the possible improvement caused by STN-DBS. Motor fluctuations, as measured by the MDS-UPDRS during the L-dopa challenge, depend on fluctuations in dopamine concentration in the nigro-striatal dopaminergic synapse that increase over time due to progressive degeneration of dopaminergic nigral neurons^68^. The prominent on–off fluctuations in later disease stages with severe degeneration are good predictors of DBS outcome^69,70^. In earlier disease stages with partially preserved dopamine buffering function of presynaptic neurons, prediction of potential benefit based on L-dopa challenge might be less reliable because of the more erratic wearing-off. This should be kept in mind when discussing DBS in patients with earlier disease stages and less pronounced motor fluctuations. In these patients, long-acting dopamine agonists may mask the true severity of untreated parkinsonism and should therefore be discontinued several days before the L-dopa challenge. This strategy was successfully used in the EARLYSTIM trial^17^, and based on this experience, we would recommend such an approach on an individual basis closely monitoring the patients in order to prevent dopamine agonist withdrawal syndrome^71^. Yet the poor fit of the linear regression shows that average disease duration is not the only factor influencing the prediction quality. It is highly likely that other unmeasured factors or unpublished data such as electrode placement or unmeasurable factors such as comorbidity also influence the quality of the prediction made by pre-operative-L-dopa challenge for the benefit of STN-DBS. Although there may be less certainty about the predictive power of the preoperative levodopa response for the benefit of STN-DBS in patients with early disease stage, the degree of motor and quality of life benefits in these patients is comparable^17^. For this reason, and as there is no loss of efficacy reported for STN-DBS in the very long term^72^, we would recommend STN-DBS in the presence of motor complications in early disease stages.

The median proportion of all surgery-related intracranial bleeding (ICH) for both targets together and regardless of whether symptomatic or not, was 3.1% in our review and 1.65% for symptomatic bleeding with permanent consequences, which is within the published ICH incidence range of 1.2–5.0%^73^. Almost exclusively in STN studies, an additional distinction was made between ICH with transient deficits (1.0%) and asymptomatic (2.1%) ICH, with the latter only documented in a few studies. Surgery-related infection was the most frequent complication of DBS with a median proportion of 5.1%. In the literature, frequencies of infectious complications have been reported in a range between 0 and 15%^73–75^, but this variation is likely due to the different definitions of a postoperative infection across centers.

Overall, the analysis of AE revealed an inconsistent and non-systematic reporting across study centers and highlights the urgent need for a uniform recording with unambiguous categories as already proposed by others^74^.

A limitation of this review is that due to the substantial numerical discrepancies between STN and GPi studies a comparative analysis between these two targets was not possible. In addition, a detailed analysis of motor complications as well as other individual variables such as surgical techniques, patient selection, and the quality of postoperative management was also not possible due to the lack of reporting or differences in the used assessment tools across centers. Nevertheless, by including also non-randomized open studies we maximized the number of studies and subjects to avoid bias and also enabled the analysis of other variables such as quality of life compared to previous meta-analyses.

In summary, DBS is an established and effective treatment for levodopa-responsive PD. While in the early days of DBS contrast ventriculography and multichannel microelectrode recording (MER) were mandatory for successful targeting, advances in imaging have now made it possible to achieve good clinical results with DBS even without MER^76^. Although our meta-analysis does not allow us to distinguish between the influences of the many individual variables of surgical techniques, patient selection, and the quality of postoperative management, it is rather surprising that the recent very convincing results of modern surgery with its technological advances^77^ are still comparable to the efficacy and safety results of the pioneering team from Grenoble^3^.

To conclude, the numerical predominance of the STN studies compared to GPi studies clearly indicates that the STN has become the preferred target for the treatment of levodopa-responsive PD.

## Methods

### Literature search and selection of articles

A comprehensive review of the literature from 1990 until August 2019 was conducted using PubMed database. Search terms included “deep brain stimulation, “neurostimulation” “Parkinson’s disease,” “subthalamic nucleus,” “globus pallidus pars interna”. The search string excluded reviews, meta-analyses, and case reports. The search was limited to articles in English language. This systematic review process and meta-analysis was performed as outlined in the PRISMA Statement (Preferred Reporting Items for Systematic Reviews and Meta-Analyses)^78^. Retrieved abstracts were reviewed, and distinct inclusion criteria were applied to the selection of articles for effectiveness and safety analysis. For the efficacy analysis, abstracts were scrutinized to include only articles with a minimum of 10 subjects reporting Unified Parkinson’s Disease Rating Scale (UPDRS) III scores at baseline and follow-up (between 6 months and 12 months after the implantation of the DBS electrodes). For more detailed information on search and selection criteria see supplementary material. After the initial review process five additional studies^3,11,17,26,36^ of seminal contribution to the clinical field of the present review were identified by the authors (P.K. and L.L) and added to the analysis, even though they did not fully comply with the search criteria as defined above. For a more detailed specification see supplementary material.

### Data extraction

The following key study characteristics and patients’ demographics were extracted: name of first author, publication year, enrollment start, and study sample size, therapeutic target location, mean age at surgery, mean disease duration, and pre-operative levodopa responsiveness. Clinical data reported before and after surgery on following variables were retrieved from the selected articles: UPDRS-II and -III score, Levodopa Equivalent Daily Dose (LEDD), dyskinesia severity, mean daily OFF time, and Parkinson’s Disease Questionnaire summary index (PDQ-39 SI). Preoperative L-dopa responsiveness (% difference between mean preoperative medication-OFF UPDRS-III score and mean medication-ON UPDRS-III score) and postoperative response to DBS (% difference between mean preoperative medications-OFF UPDRS-III score and mean postoperative medication-OFF/stimulation-ON UPDRS-III score) were calculated. The UPDRS-III score from the 6- or 12-month post-operation time points (except for Follett et al.^11^ and Schuepbach et al.^17^ for which 24-month time point was selected) was used as the postoperative UPDRS-III score. Data were aggregated regarding the different outcomes. Furthermore, a surgical efficiency index was defined

$$surgical\;efficiency = \frac{{Postoperative\;stimulation\;induced\;improvement}}{{Preoperative\;Levodopa\;induced\;improvement}}$$

For the safety analysis, the adverse events (AE) were listed if reported in at least 4 studies and were classified as surgery-related, hardware-related, an interaction of body and hardware, or in subcategories of stimulation-induced effects.

### Statistical analysis

Heterogeneity of the datasets to be analyzed was evaluated through both Cochran’s Q^79^ and Higgins I^2^ tests^80^ for the sake of relevance. Based on heterogeneity and the sample size of datasets, restricted maximum likelihood estimates of change in absolute scores/dose or percentage-of-score/dose-at-baseline after surgery (difference between postoperative stimulation-ON/medication-OFF and preoperative medication-OFF condition) were generated using the random-effects model, as suggested by Jackson and colleagues^81^.

The presence of bias was explored graphically by constructing funnel plots, with estimates of surgery effect per study plotted against the standard errors associated with these estimates. Furthermore, statistically significant evidence of bias was sought using Egger’s regression test^82^.

For STN-DBS, sample size allowed for the analysis of the influence of available baseline variables such as average disease duration, average age at implantation, UPDRS-II and -III scores, PDQ-39 SI, publication year, enrollment start, LEDD, and L-dopa responsiveness on the predictive power of preoperative L-dopa responsiveness at baseline. Normality of the distribution of datasets collected for the variables was verified through a Shapiro–Wilk test. Correlation of each dataset with the surgical efficiency index was evaluated using Pearson’s test when both datasets followed a normal distribution or Spearman’s test when one of the datasets was not following a normal distribution.

The readxl^83^ and metafor^84^ libraries were used within the R project v3.6.1^85^ (extended with RStudio v1.1.463 GUI) for statistical analysis. The Fiji^86^ software was used for linear estimation of scores on plots of publications.

### Reporting summary

Further information on research design is available in the Nature Research Reporting Summary linked to this article.

## Supplementary information

Supplementary Information

Reporting Summary

## Data Availability

The datasets used and/or analyzed during the current study are available from the corresponding author on reasonable request.
