# Supplementary material for: Subthalamic and pallidal deep brain stimulation for Parkinson’s disease—meta-analysis of outcomes
Source: NPJ Parkinsons Dis. 2021 Sep 6;7:77. doi: 10.1038/s41531-021-00223-5 (PMC8421387; doi:10.1038/s41531-021-00223-5)
Supplement: Supplementary file 1 — Supplementary Information [file 41531_2021_223_MOESM1_ESM.pdf]

Supplementary Methods

Search Parameters

The following search string was used for identification of relevant articles in the PubMed database:

((“Deep brain stimulation”) OR (DBS) OR (Neuromodulation) OR (neurostimulation)) AND ((Parkinson) OR (Parkinson’s) OR (tremor)) AND ((STN) OR (VIM) OR (GPI) OR (“subthalamic nucleus”) OR (“globus pallidus pars interna”) OR (“ventral intermediate nucleus”)) AND ((PDQ) OR (UPDRS) OR (“unified parkinson”) OR (“unified PD”) OR (“adverse event”) OR (“adverse effect”)) AND ((trial) or (study) or (series)) NOT (cZi) NOT (PPN) NOT (“treatment resistant depression”) NOT (epilepsy[MH]) NOT (dystonia[MH]) NOT (Tourette[MH]) NOT (“obsessive compulsive”) NOT (model) NOT (animal) NOT (mouse) NOT (rat) NOT (rodent) NOT (computer) NOT (acute) NOT (microelectrode) NOT (“local field”) NOT (magnetoencephalography) NOT (electromyography) NOT (electroencephalogram) NOT (metaanalysis) NOT (“meta analysis”) NOT (review[PT]) NOT (retrospective) NOT (“case report”) NOT (urinary[MH]) NOT (pilot[text]) NOT (MRI) NOT (“positron emission tomography”)AND English[Lang] AND hasabstract[text] AND "1990/01/01"[PDAT] : "2019/08/30"[PDAT]

Selection Criteria

Since the search criteria included studies with tremor and the VIM as target structure, these studies were excluded before the further review and selection process. The following inclusion and exclusion criteria were applied to the abstract citations identified in the search.

<u>Inclusion Criteria</u>: (1) Clinical article in the English language that presents either effectiveness or safety data on STN or GPI DBS for Parkinson’s Disease (PD), (2) prospective study or prospective data collection, (3) data from multiple, consistent time-points, (4) data with 6- or 12-month follow-up, (5) at least 10 subjects.

<u>Exclusion Criteria</u>: (1) Review article, meta-analysis, technique papers, editorials, letters, conference abstracts/papers/posters, and case reports, (2) retrospective data collection, (3) study on cost-effectiveness, (4) DBS for non-PD related issues (e.g. essential tremor, dystonia, obsessive compulsive disorder), (5) DBS target structure other than STN or GPI (e.g. VIM, PPN), (6) single time-point assessment, (7) inconsistent time-point data collection, (8) intraoperative evaluation, (9) single task assessment, (10) study focus on MRI or functional MRI or PET evaluation, (11) studies with < 10 subjects, (12) unilateral DBS (for efficacy analysis).

Articles meeting the criteria based on abstract review were obtained for full-text review. Final determination of article eligibility was made upon review of the full-text article by at least two

reviewers. General review articles, technique papers, editorials, letters, conference abstracts/papers/posters, and case reports were excluded. After the initial review process, five additional studies were identified by the authors (P.K. and L.L) and added to the analysis, even though they did not fully comply with the search criteria as defined above. Although the follow-up (24 months) was outside our search criteria (12 months), Follett et al. (2010) and Schüpbach et al. (2013) were added as these are two of the largest randomized controlled studies. Ford et al. (2004) were not included after the initial review process, since mean and 95% CI were only provided for the variable UPDRS-III, while no standard deviation was given for the other variables. UPDRS-III improvement in this study using blinded ratings being more modest than previously reported, we included this study because of the large impact of this study at the time of its publication and in order to avoid positive bias in selection of studies. Limousin et al. (1998) and Krack et al. (2003) were initially not selected because of possible overlaps with Obeso et al. 2001, but then included regardless, as they were some of the largest cohorts with a large impact at the time of their publication.

Supplementary Figures

Suppl. Figure 1: Schematic illustration of the literature search and study selection criteria.

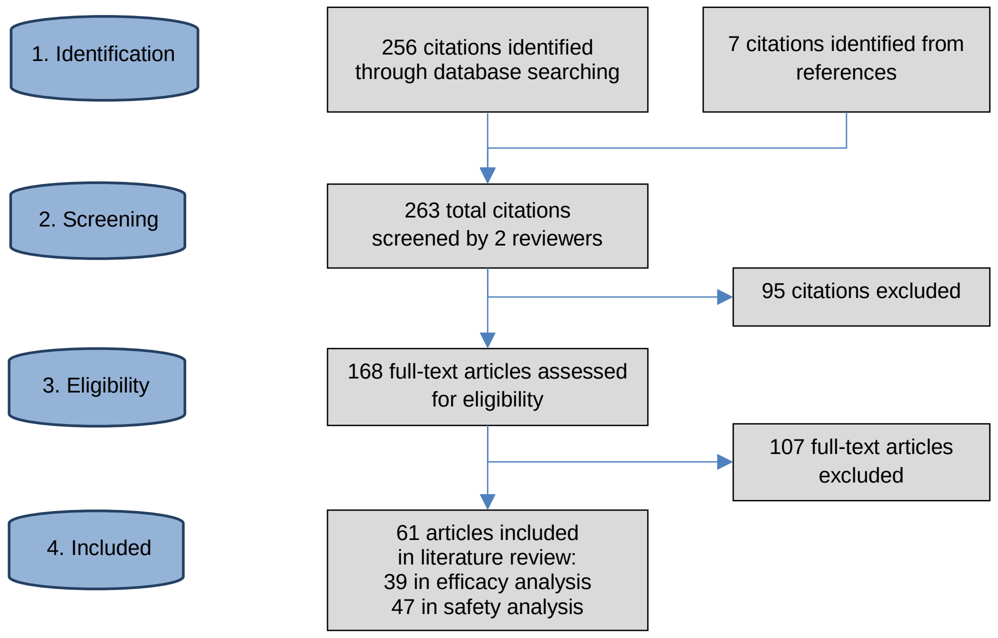

Suppl. Figure 2: Funnel plots for the variable changes analyzed.

	STN	GPi
UPDRS II	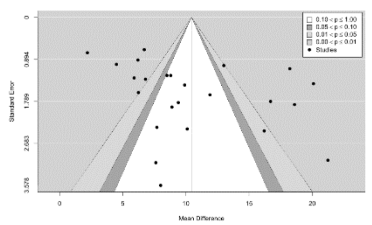	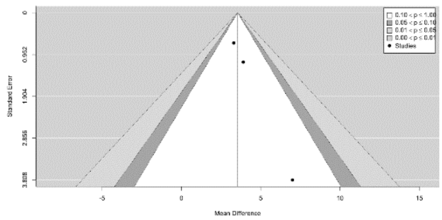
UPDRS III	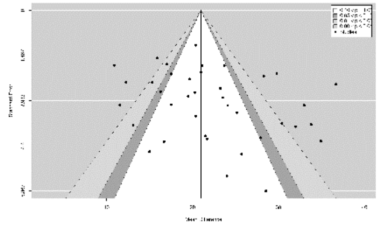	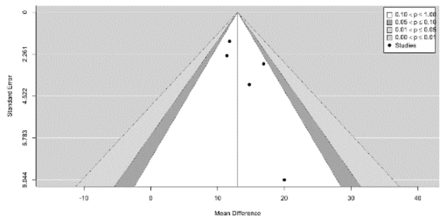
LEDD	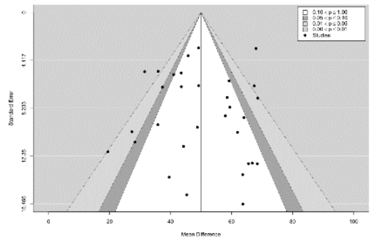	N/A
Dyskinesia	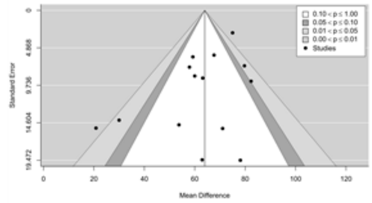	N/A
daily OFF time	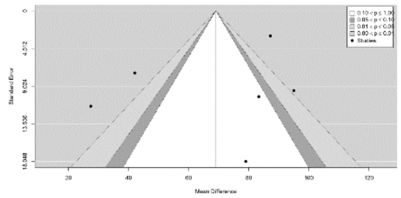	N/A
PDQ-39	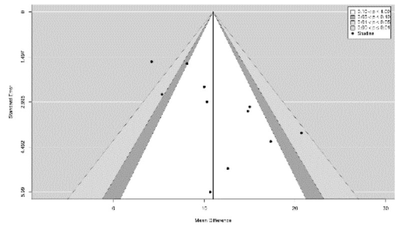	N/A

The x-axis represents the standardized mean difference and the y-axis indicates the standard error associated with each comparison. “N/A” means too scarce evidence retrieved for analysis.

Suppl. Figure 3: Dose–response relationship between preoperative L-dopa response and change in UPDRS II following STN-DBS

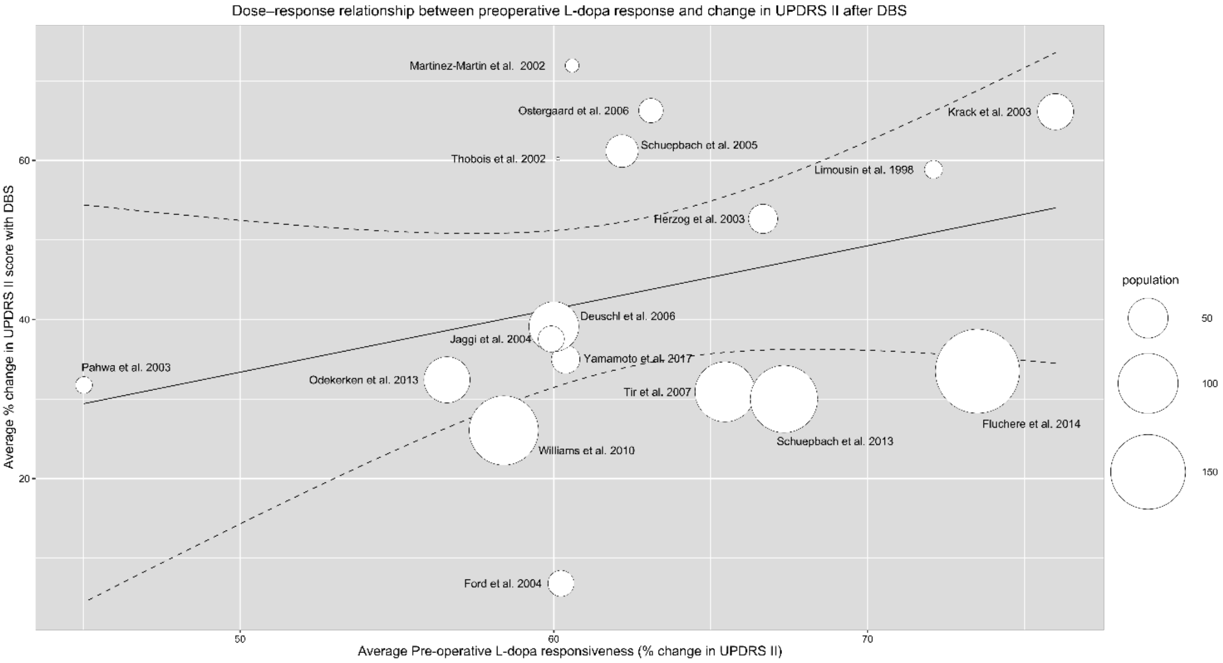

Suppl. Figure 4: Change in LEDD following STN DBS.

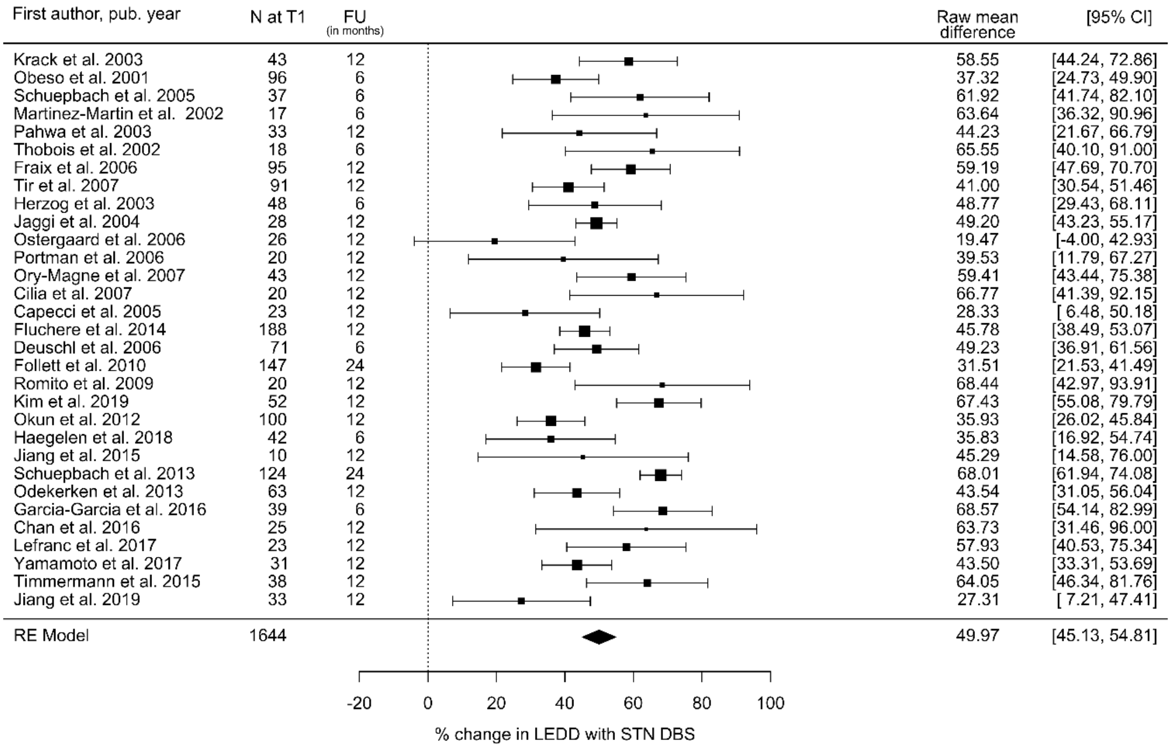

Postoperative vs. preoperative: Change in mean LEDD. N = number of subjects at follow-up; FU = follow-up time; CI = confidence interval; RE Model = random-effects model

Suppl. Figure 5: Linear regression between motor benefit prediction ratio and average disease duration prior to implant for STN-DBS patients

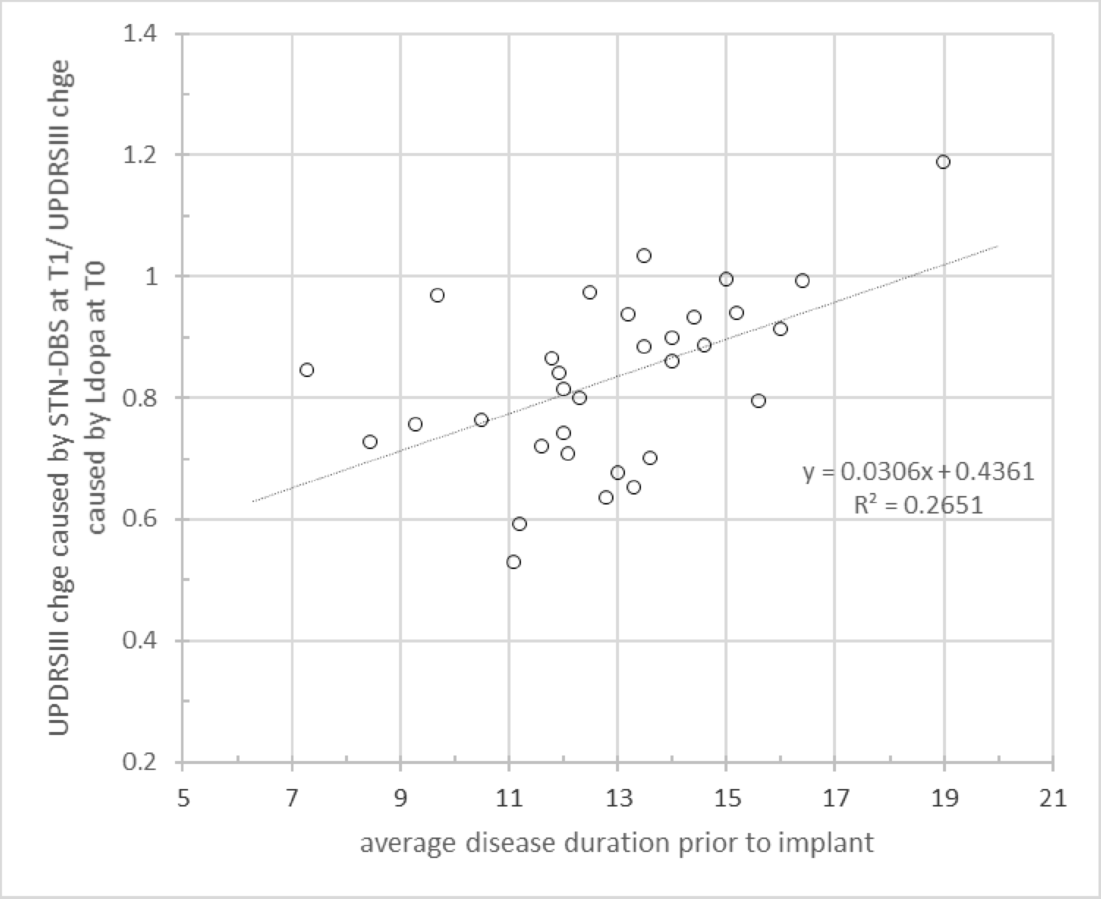

Supplementary Tables

Suppl. Table 1: Studies contributing to safety and efficacy analysis

SAFETY	EFFICACY
Capecci et al. 2005	Capecci et al. 2005
Chan et al. 2016	Chan et al. 2016
Deuschl et al. 2006	Deuschl et al. 2006
Fluchere et al. 2014	Fluchere et al. 2014
Follett et al. 2010	Follett et al. 2010
Ford et al. 2004	Ford et al. 2004
Fraix et al. 2006	Fraix et al. 2006
Garcia Ruiz et al. 2005	Garcia Ruiz et al. 2005
Herzog et al. 2003	Herzog et al. 2003
Jaggi et al. 2004	Jaggi et al. 2004
Jiang et al. 2015	Jiang et al. 2015
Lefranc et al. 2017	Lefranc et al. 2017
Martinez-Martin et al. 2002	Martinez-Martin et al. 2002
Odekerken et al. 2013	Odekerken et al. 2013
Okun et al. 2012	Okun et al. 2012
Ory-Magne et al. 2007	Ory-Magne et al. 2007
Ostergaard et al. 2006	Ostergaard et al. 2006
Portman et al. 2006	Portman et al. 2006
Romito et al. 2009	Romito et al. 2009
Schuepbach et al. 2005	Schuepbach et al. 2005
Schuepbach et al. 2013	Schuepbach et al. 2013
Thobois et al. 2002	Thobois et al. 2002
Tir et al. 2007	Tir et al. 2007
Vingerhoets et al. 2002	Vingerhoets et al. 2002
Williams et al. 2010	Williams et al. 2010
Timmerman et al. 2015	Timmerman et al. 2015
Obeso et al. 2001	Obeso et al. 2001
Birchall et al. 2017 [1]	Anderson et al. 2005
Dafsari et al. 2018 [2]	Cilia et al. 2007
Dafsari et al. 2019 [3]	Garcia-Garcia et al. 2016
Erola et al. 2006 [4]	Haegelen et al. 2018
Esselink et al. 2004 [5]	Jiang et al. 2019
Gervais-Bernard et al. 2009 [6]	Kim et al. 2019
Green et al. 2006 [7]	Krack et al. 2003
Janssen et al. 2014 [8]	Limousin et al. 1998
Krause et al. 2004 [9]	Pahwa et al. 2003
Kurcova et al. 2018 [10]	Tsai et al. 2009
Li et al. 2013 [11]	Yamamoto et al. 2017
Li et al. 2017 [12]	Yang et al. 2019
Odekerken et al. 2016	
Okun et al. 2009 [13]	
Ostergaard et al. 2002 [14]	
Rahmani et al. 2018 [15]	
Shahidi et al. 2017 [16]	
Smeding et al. 2006 [17]	
Sobstyl et al. 2017 [18]	
Tsai et al. 2013 [19]	

Data extracted from studies mentioned on orange background was used for both Efficacy and Safety analyses, data from studies on turquoise background was used in Efficacy and total ICH & infections analyses, data from studies mentioned on white background was used specifically for Efficacy or Safety analysis only.

Suppl. Table 2. Patient characteristics of efficacy studies

Authors	Pub year	Enrolment		Target	Age at surgery (years)		Disease duration before surgery (years)		LEDD (mg/day) preop			LEDD (mg/day) 6 months			LEDD (mg/day) 12 months			LEDD (mg/day) 24 months			Pre-operative levodopa response (% decrease in UPDRS III med OFF/ON)
			end year		Mean	SD [range]	Mean	SD [range]	Mean	SD	N	Mean	SD	N	Mean	SD	N	Mean	SD	N	
Limousin et al.	1998	-	-	STN	56	8	15	5	-	-	24	-	-	-	-	-	-	-	-	-	68.9
Krack et al.	2003	1993	1997	STN	55.0	7.5	14.6	5	1409	605	49	-	-	-	584	366	43	-	-	-	74.3
Obeso et al.	2001	1995	1999	STN	59.00	9.60	14.40[c]	13.09	1218.80	575.00	96	764.00	507.00	96	-	-	-	-	-	-	56.3
				GPi	55.70	9.80	14.50[c]	13.65	1090.90	543.00	38	1120.00	537.00	38	-	-	-	-	-	-	-
Vingerhoets et al.	2002	-	-	STN	63.00	8.00	16.00	5.00	1135.00	450.00	19	-	-	-	-	-	-	-	-	-	48.8
Schuepbach et al.	2005	1996	1999	STN	54.90[a]	9.10	15.20	5.30	1468.00	811.00	37	559.00	433.00	-	-	-	-	652.0	448.0	-	65.7
Martinez-Martin et al.	2002	1997	1999	STN	60.90	7.70	16.40	8.50	1400.00	658.00	17	509.00[d]	463.00	17	-	-	-	-	-	-	63.2
Pahwa et al.	2003	1997	2002	STN	58.5	[35-75]	11.8	[5.7-23.3]	10.4[f]	5.2[f]	33	-	-	-	5.8[f]	4.5[f]	33	-	-	-	37.0
Thobois et al.	2002	1998	2001	STN	56.90	6.00	13.50	4.40	1045.00	435.00	18	360.00	377.00	18	-	-	-	-	-	-	60.1
Anderson et al.	2005	1998	-	STN	61.00	9.00	15.60	5.00	100%	-	12	-	-	-	62%	-	10	-	-	-	59.2
				GPi	54.00	12.00	10.30	2.00	100%	-	11	-	-	-	97%	-	10	-	-	-	56.9
Fraix et al.	2006	1998	2002	STN	57.00	8.00	14.00	5.00	1240.00	586.00	97	-	-	-	506.00	409.00	95	-	-	-	67.5
Tir et al.	2007	1998	2003	STN	58.70	8.20	13.60	4.40	1222.00	456.00	100	-	-	-	721.00	445.00	91	-	-	-	60.0
Ford et al.	2004	1999	2001	STN	59.80	-	12.8	-	1665	-	28	-	-	-	1160	-	28	-	-	-	50.0
Herzog et al.	2003	1999	2002	STN	60.00	6.00	15.00	5.00	1425.00	843.00	48	730.00	488.00	48	820.80	-	-	458.85	-	-	57.7
Jaggi et al.	2004	1999	2002	STN	58.00	[42-77]	13.00	[4-23]	1433.00	186.00	28	858.00	140.00	28	728.00	137.00	28	-	-	-	-
Garcia Ruiz et al.	2005	-	-	STN	61.00	8.90	10.50	4.20	-	-	18	-	-	-	-	-	-	-	-	-	66.4
Ostergaard et al.	2006	-	-	STN	63.00	8.00	19.00	5.00	1197.00	532.00	26	-	-	-	964.00	501.00	26	-	-	-	54.2
Portman et al.	2006	1999	2003	STN	59.70	7.00	13.30	4.80	1242.00	678.00	20	-	-	-	751.00	398.00	20	-	-	-	50.0
Ory-Magne et al.	2007	1999	2003	STN	60.10	8.70	13.50	3.60	1466.00	665.00	45	-	-	-	595.00	437.00	43	602.0	458.0	39	59.5
Cilia et al.	2007	-	2005	STN	59.10	7.40	13.20	3.10	951.00	465.00	20	-	-	-	316.00	295.00	20	-	-	-	58.3
Capecci et al.	2005	2000	2002	STN	58.90	10.80	8.45	5.70	987.87	427.00	23	-	-	-	708.00	311.00	23	561.0	347.0	13	73.4
Williams et al.	2010	2000	2006	STN	59.00	[37-79]	11.20	[1.0-30.0]	-	-	178	-	-	-	894.00	568.00	-	-	-	-	60.3
Fluchere et al.	2014	2000	2009	STN	61.00	7.00	12.00	4.00	1173.00	495.00	213	-	-	-	636.00	376.00	188	-	-	-	74.7
Deuschl et al.	2006	2001	2004	STN	60.50	7.40	13.00[b]	5.80	1176.00	517.00	78	597.00	381.00	71	-	-	-	-	-	-	60.6
Follett et al.	2010	2002	2005	STN	61.90	8.70	11.10[b]	5.00	1295.00	585.00	147	-	-	-	-	-	-	887.0	545.0	147	47.9
				GPi	61.80	8.70	11.50[b]	5.40	1361.00	545.00	152	-	-	-	-	-	-	1118.0	562.0	152	-
Romito et al.	2009	-	2003	STN	56.40	6.90	14.30	6.20	1457.60	785.60	20	-	-	-	460.00[e]	317.00[e]	20	430.00[e]	280.00[e]	20	-
Tsai et al.	2009	2003	2007	STN	56.10	13.50	8.90	3.10	814.00	393.70	36	-	-	-	-	-	-	-	-	-	-
Kim et al.	2019	2005	2006	STN	58.70	6.30	12.50	3.80	1042.80	393.60	52	-	-	-	339.60	263.90	52	366.0	245.5	48	56.7
Okun et al.	2012	2005	2010	STN	60.60	8.30	12.10	4.90	1311.00	615.00	101	-	-	-	840.00[e]	255.00[e]	100	-	-	-	55.1
Haegelen et al.	2018	2006	2015	STN	55.60	7.60	10.10	3.80	1244.20	617.50	42	798.40	472.90	42	-	-	-	-	-	-	-
				GPi	60.50	7.20	12.80	6.20	1450.10	654.30	29	1395.40	449.40	29	-	-	-	-	-	-	-
Jiang et al.	2015	2007	2009	STN	59.40	9.30	9.30	2.90	660.40	210.10	10	-	-	-	361.30	250.90	10	-	-	-	64.6
Schuepbach et al.	2013	2006	2009	STN	52.90	6.60	7.30	3.10	935.60	239.41	124	-	-	-	-	-	-	299.30	216.03	124	62.3
Odekerken et al.	2013	2007	2011	STN	60.90	7.60	12.00	5.30	1254.00	473.00	63	-	-	-	708.00	423.00	63	-	-	-	61.7
				GPi	59.10	7.80	10.80	4.20	1331.00	637.00	62	-	-	-	1122.00	604.00	62	-	-	-	-
Garcia-Garcia et al.	2016	2007	2011	STN	58.46	8.88	11.94	4.97	1417.07	593.12	39	445.43	269.03	39	-	-	-	-	-	-	68.8
Chan et al.	2016	2009	2012	STN	55.00	6.20	13.00	7.40	1368.40	1014.00	25	-	-	-	496.30	491.00	25	602.8	650	25	-
Lefranc et al.	2017	-	-	STN	62.93	8.48	12.32	3.54	1438.40	534.26	23	-	-	-	605.10	299.56	23	-	-	-	49.5
Yamamoto et al.	2017	2009	2015	STN	66.70	5.01	11.60	20.60	1065.75	173.32	31	-	-	-	602.16	255.17	31	-	-	-	54.0
Timmermann et al.	2015	2010	2012	STN	60.20	7.80	11.70	4.60	1399.10	726.10	40	526.70	394.90	39	503.00	326.20	38	-	-	-	-
Jiang et al.	2019	2011	2014	STN	50.67	9.03	10.03	3.87	847.97	373.66	33	-	-	-	616.40	331.57	33	-	-	-	-
Yang et al.	2019	2015	2016	STN	62.81	8.47	9.70	3.90	-	-	22	-	-	-	-	-	-	-	-	-	49.0

Legend

- missing data
a, except for two patients aged 71 and 74
b, duration of use of medication for Parkinson's disease
c, duration since onset of illness
d, Treatment with L-dopa withdrawn by 4 patients 6 months after surgery.
e, estimated from quantification on publication plots using ImageJ2 (Schindelin, J.; Arganda-Carreras, I. & Frise, E. et al. (2012), "Fiji: an opensource platform for biological-image analysis", Nature methods 9(7): 676-682, PMID 22743772, doi:10.1038/nmeth.2019 (on Google Scholar).
f, expressed as levodopa equivalent dose (in LEqD units)

Suppl. Table 3: Patient characteristics in relation to the publication period (including the values of Kleiner-Fisman et al. 2006)

Publication year & therapeutic target	STN 1993 - 2004				STN 2005 - 2019				GPi 2005 - 2019			
	Weighted mean at baseline	difference between postoperative stim ON/med OFF and preoperative med OFF			Weighted mean at baseline	difference between postoperative stim ON/med OFF and preoperative med OFF			Weighted mean at baseline	difference between postoperative stim ON/med OFF and preoperative med OFF		
		Pooled mean estimate	95% CI lower limit	95% CI upper limit		Pooled mean estimate	95% CI lower limit	95% CI upper limit		Pooled mean estimate	95% CI lower limit	95% CI upper limit
Age at operation (years)	58.21/58.52	-	58.06/58.30	58.36/58.74	59.06	-	58.91	59.21	60.65	-	52.04	69.27
Disease duration (years)	13.38/14.21	-	13.15/14.08	13.60/14.34	11.81	-	11.71	11.91	11.43	-	9.80	13.05
UPDRS II	N/A/25.82	13.35 (49.9%/51.7%)	10.85 (43.0%/42.0%)	15.85 (56.9%/61.4%)	21.57	8.79 (40.9%)	6.66 (31.0%)	10.91 (50.8%)	19.19	3.55 (18.5%)	2.41 (12.6%)	4.68 (24.4%)
UPDRS III	N/A/49.35	27.55 (52.0%/55.8%)	24.23 (48.1%/49.1%)	30.87 (56.5%/62.6%)	42.55	20.93 (49.5%)	18.78 (44.4%)	23.07 (54.6%)	42.65	12.13 (28.4%)	9.73 (22.8%)	14.53 (34.1%)

$weighted\ mean = \frac{\sum_{i=1}^{N}(x_i \times n_i)}{\sum_{i=1}^{N} n_i}$; values in grey were calculated from the data published in Kleiner-Fisman et al. 2006; percentages refer to the weighted mean score at baseline.

Suppl. Table 4: Quantification and significance of funnel plot asymmetry

	STN	GPi
UPDRS II	z = 1.4386 P = 0.1503	z = 1.0227 P = 0.3064
UPDRS III	z = 1.7059 P = 0.0880	z = 1.3660 P = 0.1719
LEDD	z = 0.2934 P = 0.7692	N/A
Dyskinesia	z = -1.7919 P = 0.0731	N/A
daily OFF time	z = -0.2121 P = 0.8320	N/A
PDQ-39	z = 2.9853 **P = 0.0028**	N/A

"P" means p-value; "z" is the statistics obtained from a z-test on the regression.

Suppl. Table 5: Proportions of surgery-, hardware-, disease-, stimulation-, and therapy-related side effects

Event type	STN			GPi			All		
	Median proportion in %	nb studies	total sample size	Median proportion in %	nb studies	total sample size	Median proportion in %	nb studies	total sample size
Surgery related									
ICH, Total	3.45	25	1739	2.17	4	281	3.12	25	2020
ICH, permanent effect	1.80	22	1431	2.17	2	175	1.65	22	1519
ICH, temporary effect	0.97	17	1285	0.00	1	152	0.97	17	1350
ICH, asymptomatic	2.56	7	369		-	-	2.08	7	457
Infection (surgery or < 3months)	4.54	25	1829	5.48	4	281	5.13	25	2110
Infection (not surgery, > 3 months)	1.47	11	791	0.00	1	47			
Seizures	1.12	13	853	1.54	2	112			
Hemiparesis secondary to ICH	2.56	5	400		-	0			
Wound healing	3.90	4	523		-	0			
lead bad placement	2.79	14	1034		-	0			
Lead migration	1.25	8	815	2.13	1	47			
Stroke	1.80	4	267	0.00	1	152			
pain / discomfort / erythema at sight of incision	13.64	1	22	4.35	1	23			
wire tightness		-	-		-	-			
other (surgery related)		-	-		-	-			
Paraventricular or subdural Hematoma	2.47	2	81		-	-			
Interaction body- hardware									
Infection (late, not surgery related): see in surgery-related section	3.96	1	101		-	-			
Subcutan. hematoma or seroma	2.56	11	812		-	-			
Skin Erosion	1.94	7	458		-	-			

Hardware related									
Broken lead	2.84	8	605		-	-			
Dislocation (move) of IPG	4.10	4	408		-	-			
DBS System (unspecified)	3.28	6	634	1.32	1	152			
unexplained device switch off	5.00	1	20		-	-			
lead extension malfunction		-	-		-	-			
Death									
Death after hemorrhage		15	1316	0.00	4	287			
linked to surgery (other than ICH)		15	1127	0.00	4	287			
Death (by suicide)		13	1096	0.00	4	287			
Death linked to therapy		11	990	0.00	4	287			
Death (unrelated to DBS)	0.56	15	1090	0.00	4	287			
Death (origin not assigned)	4.55	2	123	0.00	4	287			
Total deaths reported	2.50	25	2531	0.00	4	287			
Speech disorder									
Dysarthria	10.13	22	1363	14.62	2	112			
Dysphasia	5.07	4	282	5.38	2	112			
Speech problem	13.38	8	377	40.23	2	175			
aphasia	2.38	1	42		-	-			
sum of speech disorders	15.38	25	1555	34.14	4	287			
Worsening of motor impairment / PD symptoms									
General motor impairment / rebound of symptoms	6.74	5	602	23.68	1	152			
Dyskinesia	12.50	15	1182	23.03	3	264			
Worse motor fluctuations	8.33	5	540	8.70	1	23			
Abnormal gait	8.82	11	803	26.99	2	175			
Balance disorder	8.09	9	520	16.52	4	287			
Freezing	17.39	5	252	15.79	2	175			
Falls	6.42	10	961	41.45	1	152			
Akinesia/bradykinesia	21.77	1	147	23.68	1	152			
Dystonia, rigidity	12.91	6	400	15.53	2	175			
Eye disorder	2.50	7	429	5.68	2	112			
Worsening of mobility	14.20	2	221		-	-			
hyperkinesia	7.41	1	27		-	-			
paralysis		-	-		-	-			
unsatisfactory results	5.56	1	18		-	-			
Psychiatry									
Depression	9.95	20	1294	29.69	2	175			
Suicide attempt	1.15	10	1110	0.66	1	152			
Suicide ideation	0.50	4	550	2.50	2	175			

Confusion	5.06	22	1566	23.57	2	175			
Anxiety	3.90	8	756	30.43	1	23			
Apathy / fatigue	9.35	10	559	13.04	1	23			
Hallucinations	3.85	9	594	4.35	1	23			
Psychosis	2.11	9	978		-	-			
Delirium / Delusions	11.90	2	106	10.77	2	112			
Emotional lability	14.65	6	305	36.15	2	112			
Hypersexuality	4.00	7	264	4.35	1	23			
Impulse control	2.97	7	543		-	-			
Hypomania	4.35	9	569		-	-			
Manic Tendencies	15.00	3	59	4.35	1	23			
decreased libido	13.64	1	22	21.74	1	23			
Alcohol abuse	2.00	1	50		-	-			
Dopamine withdrawal syndrom	6.72	2	137		-	-			
anorexia	4.55	1	22	13.04	1	23			
Anhedonia	10.00	1	20		-	-			
agitation	15.00	1	20		-	-			
Punding	8.70	1	23		-	-			
Cognitive / behavioural									
Dementia	10.81	2	68		-	0			
Cognitive decline	2.45	10	788	7.84	2	175			
Sleep disturbance	7.35	5	503	47.83	1	23			
Apraxia of eyelid opening	7.47	12	578	8.46	2	112			
Aggressive behavior	8.11	3	179	21.74	1	23			
OCD	22.73	1	22	8.70	1	23			
Temporary altered mentation	5.00	1	20		-	-			
Sensory impairment									
Paresthesia	3.37	4	284	0.77	2	112			
Sensory disturbance	7.05	4	168	4.35	3	135			
Diplopia	2.69	4	266		-	0			
sum of sensory	5.15	9	590	4.35	3	135			
Body reactions									
Respiratory (pneumonia)	2.72	11	891	5.26	1	152			
Cardiovascular	2.04	7	576	2.50	2	175			
Gastrointestinal	2.37	6	692	2.83	2	175			
Injury	6.45	1	124		-	-			
Musculo-skeletal	6.47	6	570	6.32	2	175			
Urogenital	4.41	3	336	13.04	1	23			
Erectile/sexual dysfunction	3.64	2	244		-	-			
Weight change	25.68	10	413	8.70	1	23			
Hypersalivation	5.10	8	404	11.83	2	112			
Dysphagia	4.50	6	208	11.06	2	112			

Renal and urinary disorder	0.91	2	221		-	-			
Air embolism (circulatory)		-	-	4.35	1	23			
Pulmonary embolism	4.35	5	153		-	-			
breath shortness	4.55	1	22		-	-			
suffocation	5.00	3	69		-	-			
hyperhidrosis	13.38	2	49		-	-			
nausea	6.82	2	123	4.35	1	23			
dry mouth	9.09	1	22	4.35	1	23			
weakness	18.18	1	22	4.35	1	23			
Orthostasis / dizziness	18.18	1	22	8.70	1	23			
Lower extremity edema	4.55	1	22		-	-			
Lower limbs/extremity phlebitis	7.94	2	60		-	-			
Others									
Pain	3.68	7	759	8.70	1	23			
Headache	6.75	2	158	13.04	1	23			
Hiccups	1.59	2	106	7.69	2	112			
Syncope	2.72	1	147	0.66	1	152			
Hernia	1.36	1	147	2.83	2	175			
Bone fracture	6.56	2	74	8.70	1	23			
Skin Disorder	2.50	4	385	8.70	1	23			
Influenza / bronchitis	3.68	3	222	4.35	1	23			
Stroke non DBS	3.33	3	187	0.00	1	152			
Unclassified or "other"	10.00	13	1146	17.02	3	264			
CSF leakage	3.78	2	59		-	-			
cold/ low grade fever	9.55	2	32	4.35	1	23			
Pneumocephalus	5.71	3	72		-	-			
swollen lymph node	4.55	1	22		-	-			
chest pain	9.55	2	32		-	-			
veinous hemorrhage (symptomatic)		-	-	8.70	1	23			
transient ischemic attack		-	-		-	-			
Arthritis	8.69	2	62		-	-			
Polyneuropathy	0.00	1	101		-	-			

Note: No distinction was made between different types of ICH in the data extracted from Obeso et al. 2001 and Follett et al. 2010. Hence, ICH data reported from these articles were only included in the overall ICH proportions (including STN and GPi for all kinds of ICH).

Suppl. Table 6: Analysis of correlation between STN-DBS benefit prediction quality based on UPDRS III and different variables.

Variable at baseline	Average disease duration	Average age at implant	UPDRS III at baseline	UPDRS II at baseline	PDQ-39 at baseline	Pub year	Enrolment start	LEDD at baseline
Pearson correlation coefficient	0.514 **(P= 0.002568)**	-0.131 (P=0.474)	0.292 (P=0.1109)	0.359 (P=0.131)	N/A	N/A	N/A	-0.046 (P=0.823)
Spearman correlation coefficient	N/A	N/A	N/A	N/A	-0.452 (P=0.268)	-0.279 (P=0.122)	-0.317 (P=0.114)	N/A

Supplementary References

1 Birchall, E. L. *et al.* The effect of unilateral subthalamic nucleus deep brain stimulation on depression in Parkinson's disease. *Brain stimulation* **10**, 651-656, doi:10.1016/j.brs.2016.12.014 (2017).

2 Dafsari, H. S. *et al.* Quality of life outcome after subthalamic stimulation in Parkinson's disease depends on age. *Movement disorders : official journal of the Movement Disorder Society* **33**, 99-107, doi:10.1002/mds.27222 (2018).

3 Dafsari, H. S. *et al.* EuroInf 2: Subthalamic stimulation, apomorphine, and levodopa infusion in Parkinson's disease. *Movement disorders : official journal of the Movement Disorder Society* **34**, 353-365, doi:10.1002/mds.27626 (2019).

4 Erola, T. *et al.* Efficacy of bilateral subthalamic nucleus (STN) stimulation in Parkinson's disease. *Acta neurochirurgica* **148**, 389-394, doi:10.1007/s00701-005-0662-8 (2006).

5 Esselink, R. A. *et al.* Unilateral pallidotomy versus bilateral subthalamic nucleus stimulation in PD: a randomized trial. *Neurology* **62**, 201-207, doi:10.1212/01.wnl.0000103235.12621.c3 (2004).

6 Gervais-Bernard, H. *et al.* Bilateral subthalamic nucleus stimulation in advanced Parkinson's disease: five year follow-up. *Journal of neurology* **256**, 225-233, doi:10.1007/s00415-009-0076-2 (2009).

7 Green, A. L. *et al.* STN vs. Pallidal Stimulation in Parkinson Disease: Improvement With Experience and Better Patient Selection. *Neuromodulation : journal of the International Neuromodulation Society* **9**, 21-27, doi:10.1111/j.1525-1403.2006.00038.x (2006).

8 Janssen, M. L. *et al.* Subthalamic nucleus high-frequency stimulation for advanced Parkinson's disease: motor and neuropsychological outcome after 10 years. *Stereotactic and functional neurosurgery* **92**, 381-387, doi:10.1159/000366066 (2014).

9 Krause, M., Fogel, W., Mayer, P., Kloss, M. & Tronnier, V. Chronic inhibition of the subthalamic nucleus in Parkinson's disease. *Journal of the neurological sciences* **219**, 119-124, doi:10.1016/j.jns.2004.01.004 (2004).

10 Kurcova, S. *et al.* Bilateral subthalamic deep brain stimulation initial impact on nonmotor and motor symptoms in Parkinson's disease: An open prospective single institution study. *Medicine* **97**, e9750, doi:10.1097/MD.0000000000009750 (2018).

11 Li, D. *et al.* Subthalamic nucleus deep brain stimulation for Parkinson's disease: 8 years of follow-up. *Translational neurodegeneration* **2**, 11, doi:10.1186/2047-9158-2-11 (2013).

12 Li, D. *et al.* Remotely Programmed Deep Brain Stimulation of the Bilateral Subthalamic Nucleus for the Treatment of Primary Parkinson Disease: A Randomized Controlled Trial Investigating the Safety and Efficacy of a Novel Deep Brain Stimulation System. *Stereotactic and functional neurosurgery* **95**, 174-182, doi:10.1159/000475765 (2017).

13 Okun, M. S. *et al.* Cognition and mood in Parkinson's disease in subthalamic nucleus versus globus pallidus interna deep brain stimulation: the COMPARE trial. *Annals of neurology* **65**, 586-595, doi:10.1002/ana.21596 (2009).

14 Ostergaard, K., Sunde, N. & Dupont, E. Effects of bilateral stimulation of the subthalamic nucleus in patients with severe Parkinson's disease and motor fluctuations. *Movement disorders : official journal of the Movement Disorder Society* **17**, 693-700, doi:10.1002/mds.10188 (2002).
15 Rahmani, M. *et al.* Deep Brain Stimulation in Moroccan Patients With Parkinson's Disease: The Experience of Neurology Department of Rabat. *Frontiers in neurology* **9**, 532, doi:10.3389/fneur.2018.00532 (2018).
16 Shahidi, G. A. *et al.* Outcome of subthalamic nucleus deep brain stimulation on long-term motor function of patients with advanced Parkinson disease. *Iranian journal of neurology* **16**, 107-111 (2017).
17 Smeding, H. M. *et al.* Neuropsychological effects of bilateral STN stimulation in Parkinson disease: a controlled study. *Neurology* **66**, 1830-1836, doi:10.1212/01.wnl.0000234881.77830.66 (2006).
18 Sobstyl, M. *et al.* Unilateral Subthalamic Nucleus Stimulation in the Treatment of Asymmetric Parkinson''s Disease with Early Motor Complications. *Turkish neurosurgery* **27**, 294-300, doi:10.5137/1019-5149.JTN.14894-15.0 (2017).
19 Tsai, S. T. *et al.* Long-term outcome of young onset Parkinson's disease after subthalamic stimulation--a cross-sectional study. *Clinical neurology and neurosurgery* **115**, 2082-2087, doi:10.1016/j.clineuro.2013.07.014 (2013).
