# Supplementary material for: Subthalamic and pallidal deep brain stimulation for Parkinson’s disease—meta-analysis of outcomes
Source: NPJ Parkinsons Dis. 2021 Sep 6;7:77. doi: 10.1038/s41531-021-00223-5 (PMC8421387; doi:10.1038/s41531-021-00223-5)
Supplement: Supplementary file 2 — Reporting Summary [file 41531_2021_223_MOESM2_ESM.pdf]

nature research

Corresponding author(s): NPJPARKD-00625

Last updated by author(s): Apr 7, 2021

Reporting Summary

Nature Research wishes to improve the reproducibility of the work that we publish. This form provides structure for consistency and transparency in reporting. For further information on Nature Research policies, see our Editorial Policies and the Editorial Policy Checklist.

Statistics

For all statistical analyses, confirm that the following items are present in the figure legend, table legend, main text, or Methods section.

n/a	Confirmed	
☐	☒	The exact sample size (n) for each experimental group/condition, given as a discrete number and unit of measurement
☐	☒	A statement on whether measurements were taken from distinct samples or whether the same sample was measured repeatedly
☐	☒	The statistical test(s) used AND whether they are one- or two-sided<br>*Only common tests should be described solely by name; describe more complex techniques in the Methods section.*
☐	☒	A description of all covariates tested
☐	☒	A description of any assumptions or corrections, such as tests of normality and adjustment for multiple comparisons
☐	☒	A full description of the statistical parameters including central tendency (e.g. means) or other basic estimates (e.g. regression coefficient) AND variation (e.g. standard deviation) or associated estimates of uncertainty (e.g. confidence intervals)
☐	☒	For null hypothesis testing, the test statistic (e.g. F, t, r) with confidence intervals, effect sizes, degrees of freedom and P value noted<br>*Give P values as exact values whenever suitable.*
☒	☐	For Bayesian analysis, information on the choice of priors and Markov chain Monte Carlo settings
☐	☒	For hierarchical and complex designs, identification of the appropriate level for tests and full reporting of outcomes
☐	☒	Estimates of effect sizes (e.g. Cohen's d, Pearson's r), indicating how they were calculated

Our web collection on statistics for biologists contains articles on many of the points above.

Software and code

Policy information about availability of computer code

Data collection	*Provide a description of all commercial, open source and custom code used to collect the data in this study, specifying the version used OR state that no software was used.*
Data analysis	The readxl and metafor libraries were used within the R project v3.6.131 (extended with RStudio v1.1.463 GUI) for statistical analysis. The Fiji software was used for linear estimation of scores on plots of publications. The estimation of Tau^2 was obtained using the "Restricted Maximum Likelihood (REML) method.

For manuscripts utilizing custom algorithms or software that are central to the research but not yet described in published literature, software must be made available to editors and reviewers. We strongly encourage code deposition in a community repository (e.g. GitHub). See the Nature Research guidelines for submitting code & software for further information.

Data

Policy information about availability of data

All manuscripts must include a data availability statement. This statement should provide the following information, where applicable:

- Accession codes, unique identifiers, or web links for publicly available datasets
- A list of figures that have associated raw data
- A description of any restrictions on data availability

The datasets used and/or analysed during the current study are available from the corresponding author on reasonable request.

Field-specific reporting

Please select the one below that is the best fit for your research. If you are not sure, read the appropriate sections before making your selection.

☒ Life sciences ☐ Behavioural & social sciences ☐ Ecological, evolutionary & environmental sciences

For a reference copy of the document with all sections, see nature.com/documents/nr-reporting-summary-flat.pdf

Life sciences study design

All studies must disclose on these points even when the disclosure is negative.

Sample size	Does not apply since this is a meta-analysis
Data exclusions	A comprehensive review of the literature from 1990 until August 2019 was conducted using PubMed database. Search terms included “deep brain stimulation, “neurostimulation” “Parkinson’s disease,” “subthalamic nucleus,” “globus pallidus pars interna”. The search string excluded reviews, meta-analyses, and case reports. The search was limited to articles in English language. This systematic review process and meta-analysis was performed as outlined in the PRISMA Statement (Preferred Reporting Items for Systematic Reviews and Meta-Analyses). Retrieved abstracts were reviewed, and distinct inclusion criteria were applied to the selection of articles for effectiveness and safety analysis.
Replication	Does not apply since this is a meta-analysis
Randomization	Does not apply since this is a meta-analysis
Blinding	Does not apply since this is a meta-analysis

Reporting for specific materials, systems and methods

We require information from authors about some types of materials, experimental systems and methods used in many studies. Here, indicate whether each material, system or method listed is relevant to your study. If you are not sure if a list item applies to your research, read the appropriate section before selecting a response.

Materials & experimental systems

n/a	Involved in the study	
☒	☐	Antibodies
☒	☐	Eukaryotic cell lines
☒	☐	Palaeontology and archaeology
☒	☐	Animals and other organisms
☒	☐	Human research participants
☒	☐	Clinical data
☒	☐	Dual use research of concern

Methods

n/a	Involved in the study	
☒	☐	ChIP-seq
☒	☐	Flow cytometry
☒	☐	MRI-based neuroimaging
